# Predictors of exclusive breastfeeding: observations from the Alberta pregnancy outcomes and nutrition (APrON) study

**DOI:** 10.1186/1471-2431-13-77

**Published:** 2013-05-16

**Authors:** Mahsa Jessri, Anna P Farmer, Katerina Maximova, Noreen D Willows, Rhonda C Bell

**Affiliations:** 1 Department of Nutritional Sciences, Faculty of Medicine, University of Toronto, Toronto, ON M5S 3E2, Canada; 2 Human Nutrition Division, Department of Agricultural, Food and Nutritional Sciences, University of Alberta, Edmonton, AB, Canada; 3 The Center for Health Promotion Studies, University of Alberta, Edmonton, AB, Canada; 4 Department of Public Health Sciences, University of Alberta, Edmonton, AB, Canada; 5 Human Nutrition Division, Alberta Institute for Human Nutrition, and Center for Health Promotion Studies, Edmonton Clinic Health Academy 4-370, University of Alberta, Edmonton, AB T6G 1C9, Canada

**Keywords:** Exclusive breastfeeding, Predictors, Canada, Alberta pregnancy outcomes, Nutrition study

## Abstract

**Background:**

Despite growing evidence that supports the importance of 6-month exclusive breastfeeding, few Canadian mothers adhere to this, and early weaning onto solids is a common practice. This study assessed infant feeding transitions during the first 6 months postpartum and factors that predicted exclusive breastfeeding to 3 and 6 months.

**Methods:**

This prospective cohort study was part of the Alberta Pregnancy Outcomes and Nutrition study (APrON). From an initial sample of 600 pregnant women recruited from Edmonton and Calgary, 402 mothers provided complete details at 3 months postpartum; 300 stayed on to provide information at 6 months postpartum. During pregnancy and at 3 and 6 months postpartum, data on maternal and infant socio-demographic, behavior, and feeding were collected.

**Results:**

Even though there was a high rate of “ever having breastfed” (98.6%), exclusive breastfeeding rates for 3 and 6 months were 54.0% and 15.3%, respectively. After controlling for potential confounders, the study showed that mothers who held post-graduate university degrees were 3.76 times more likely to breastfeed exclusively for 6 months than those without a university degree (95% CI: 1.30-10.92; p = 0.015). In addition, mother of previous children were more likely to breastfeed exclusively for 6 months (OR: 2.21, 95% CI: 1.08-4.52; p = 0.031). Mothers who were in the highest quartile of the Iowa Infant Feeding Attitude Score were 4.29 and 5.40 times more likely to breastfeed exclusively for 3 months (95% CI: 1.31-14.08; p-trend < 0.001) and 6 months (95% CI: 2.75-10.60; P-trend < 0.001), respectively.

**Conclusions:**

The 6-month exclusive breastfeeding rate in Alberta is considerably below national and international breastfeeding recommendations. Professional advice that focuses on prenatal maternal knowledge, attitudes, and misperceptions may promote adherence to World Health Organization breastfeeding guidelines. Knowing that exclusive breastfeeding is less likely to take place among lower-educated, primiparous women may help health practitioners focus their support and education for this group.

## Background

Breastfeeding has been shown to be an unsurpassed means of infant feeding that provides many health benefits to both infants [1-9] and mothers [4,10]. Breastfeeding may help prevent chronic diseases and conditions such as childhood obesity [11], type 2 diabetes [12], and asthma [4]. Exclusive breastfeeding for 6 months confers several health benefits, such as decreased rates of gastrointestinal tract infections and morbidity [13,14]. Breastfeeding mothers may have delayed fertility, lower risk of postpartum bleeding, faster return to pre-pregnancy weight, and decreased risk of breast and ovarian cancers [4,15-17]. The short-term health risks associated with feeding exclusively by formula (i.e., not breastfeeding at all) include increased risk of otitis media and diarrhea [18], increased susceptibility to rare diseases, such as leukemia [4,19], severe lower respiratory tract infections [4,20], and sudden infant death syndrome [4].

In 2004 the Public Health Agency of Canada, the Canadian Paediatric Society, and Health Canada [21,22] endorsed the 2001 World Health Organization (WHO) breastfeeding guidelines that recommend that all infants be exclusively breastfed for the first 6 months of life with continued breastfeeding for 2 years and beyond [14,17]. Although the majority of Canadian women initiate breastfeeding, early supplementation of infants’ diets [23-26] means that only a minority of women exclusively breastfeed for 6 months. In 2003, a national Canadian survey reported that 81% of infants were fed solid foods before 3 months of age, and 89% and 100% were fed solids before 5 and 6 months of age, respectively [27]. Other Canadian studies reported that exclusive breastfeeding is commonly practiced among highly educated, multiparous, older women who live with a partner, have lower body mass indices (BMIs), and reside in urban areas [23,24,28]. Other studies identify maternal attitude as a better predictor of infant feeding decisions than socio-demographic factors [29-32]. According to the “Theory of Reasoned Action,” an individual’s intention to perform a behaviour is the primary determinant of the behaviour [33], which is itself influenced by the individual’s attitude toward performing the behaviour [34]. Maternal attitude may be a more suitable focus for study than fixed socio-economic factors [35].

Understanding the predictors of exclusive breastfeeding may assist in the creation of programs that promote breastfeeding exclusivity and may lead to more infants being exclusively breastfed in accordance with the WHO guidelines [23]. Gaps and limitations of previous infant feeding studies, changes over time in infant feeding practices and guidelines [14,17,36,37], and the differences in provincial breastfeeding rates in Canada [23] suggest that more understanding of infant feeding patterns is required. There are no previous Canadian studies that assess the psychosocial determinants of exclusive breastfeeding and infant feeding transitions during the first 6 months postpartum.

The aims of this study were to evaluate in a longitudinal birth cohort a) the transitions in infant feeding practices between 3 and 6 months postpartum relative to the current WHO and Health Canada guidelines and b) the relationship between parental/infant characteristics and exclusive breastfeeding to 3 and 6 months after birth.

## Methods

### Population and sampling

This study is part of the Alberta Pregnancy Outcomes and Nutrition (APrON) study, the largest on-going Canadian prenatal nutrition cohort study that uses repeated measures over a 4-year period to assess health and nutrition outcomes among pregnant women and their children. (See http://www.apronstudy.ca and Kaplan et al. 2012 for more information on rationale and methods) [38]. During the first phase of the APrON study (May 2009-March 2010), 600 pregnant women from Edmonton and Calgary, Alberta (Western Canada), were recruited through media advertising and contact in maternity clinics. Pregnant women who delivered a baby were followed postpartum. The intake criteria were: 1) resident of Calgary/Edmonton or surrounding areas, 2) reside in the region for at least 6 months, 3) ≤27 weeks gestation, 4) ≥16 years of age, 5) able to speak and write in English, and 6) able to complete consent forms for themselves and their infants.

**Figure 1:**
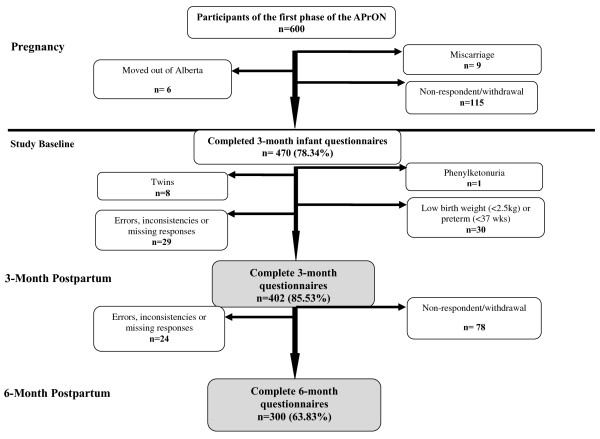
**Recruitment details: reasons for non-participation and non-eligibility among a subsample of participants from the first phase of Alberta Pregnancy Outcomes and Nutrition (APrON) study.** (legend) ^*^Examples of inconsistencies in responses include mothers who had indicated they have “stopped breastfeeding” or “have not yet introduced breast milk”, but at the same time provided “positive frequencies” for breast milk feeding in the FFQs. Other examples include mothers who had stated they have “never breastfed their infants” while providing “age of introduction” or “intake frequency” of breast milk. In addition, some mothers indicated they had “not yet introduced breast milk” or had “stopped breastfeeding” while they also stated on another questionnaire that they were “currently breastfeeding”.

Of the 600 pregnant women who went through the intake process in the first cohort of the APrON, 470 were followed up with and completed the 3-month infant surveys (response rate = 78.34%) (Figure 1). Infants who were healthy (without medical conditions or congenital malformations), singleton, term (>37 weeks gestational age), and with normal birth weight (>2500 grams at birth) were included. Records with inconsistencies, errors, or missing values for infant feeding variables at 3- and 6-month time points were excluded. There remained 402 and 300 infant records available for analyses at 3 and 6 months of age, respectively (Figure 1).

General characteristics (e.g., age, BMI, parity, IIFAS scores, and parental education level) of participants who were included in the final analyses (n = 300) were similar to the characteristics of the initial participants (n = 600).

The Health Research Ethics Board at the University of Alberta and the Conjoint Health Research Ethics Board at the Faculty of Medicine, University of Calgary, approved the study. Prior to the study, participants provided informed written consent for their own and their infants’ participation.

### Measurements

#### Baseline assessments

Several baseline variables collected during pregnancy were considered as possible predictors of exclusive breastfeeding. Parental socio-demographic and lifestyle characteristics obtained from self-report questionnaires were: age (in years), marital status, educational level, occupational status, annual household income, household size, birthplace, gravida, parity, chronic disease history, medication use, and pre-pregnancy weight. At the first prenatal visit, trained research staff measured the women’s height using a digital stadiometer (Charder HM200P Portstad Portable Stadiometer, USA).

#### Iowa infant feeding attitude scale (IIFAS)

During the third trimester, women completed the IIFAS survey to determine their knowledge and attitudes toward breastfeeding [30]. The scale developer (A. de la Mora) [30] granted permission to use the tool. The IIFAS is a self-report tool composed of 17 items on a 5-point Likert scale ranging from “strongly disagree” to “strongly agree”. Items that favoured formula feeding were reverse-coded, and a summative score of all items made up the total IIFAS score [30]. The overall scores were organized into 3 categories: positive to breastfeeding (70–85), neutral (49–69), and positive to formula feeding (17–48) [29,30]. Previous studies that have evaluated the IIFAS, this tool has shown strong internal consistency and adequate construct, content, and predictive validity among a variety of populations [39].

We assessed the reliability (internal consistency) and validity of the IIFAS in this sample of pregnant women. The IIFAS had robust internal reliability (Cronbach’s α = 0.81) (cut-off point = 0.70). The reliability testing showed that all but two of the IIFAS items had positive, significant corrected item-total correlations (>0.2) – “breast milk is lacking in iron” and “a mother who occasionally drinks alcohol should not breastfeed her baby” [30] – and were left in the analyses due to the overall strong reliability of the IIFAS.

#### Follow-up assessments

At the 3-month postpartum visit, mothers were weighed to the nearest 0.01 kg using digital scales (Healthometer Professional 98 752KL, Pelstar LLC, IL, USA). Infant characteristics such as gender, gestational age, birth weight, number of siblings, birth problems, medical conditions, and feeding behaviours were collected through hospital charts and questionnaires. Infants were weighed at 3 months postpartum through direct measurement. Mothers completed a “Child Food and Liquid Intake” (CFLI) questionnaire at approximately 3 and 6 months postpartum (retrospective recall). The APrON team developed the questionnaire based on 24-hour dietary recalls from Canadian children (S. Atkinson, personal communication) and other large surveys of infants and toddlers [40-42]. A diverse group of health professionals (i.e., dietitians, pediatricians, midwives, nurses, lactation consultants, and public health nurses involved in well-baby clinics and home care) evaluated both the content validity and face validity of the questionnaire. The CFLI included structured questions related to 1) breastfeeding and formula feeding practices and the reasons for breastfeeding cessation (6 questions), 2) the age of initiation of each food item and other infant feeding practices and behaviours (e.g., vitamin/mineral supplementation and feeding on demand/scheduled feeding), and 3) a 40-item qualitative food frequency questionnaire (FFQ). The infant FFQ was pre-tested in a group of women whose infants were between 3 and 36 months of age (n = 10/age group), and the results showed that the tool was able to detect significant dietary changes between each separate age group.

### Classification of infant feeding patterns

Infants’ feeding patterns were classified according to the WHO definitions: “exclusive breastfeeding”, “predominant breastfeeding”, “complementary/replacement feeding”, and “not breastfeeding” [43]. Exclusive breastfeeding to 6 months, which is the primary feeding pattern of interest in this study, is defined as the intake of breast milk (e.g., directly, expressed, or from a wet nurse) without any additional liquids or solid/semi-solid foods [44]; intakes of oral rehydration solution (ORS), vitamins, minerals, and medications in the form of drops or syrups are allowed [43]. Predominant breastfeeding is defined as the intake of breast milk as the primary source of nourishment [43] along with water, water-based drinks, fruit juice, or ritual fluids; ORS, vitamins, minerals, and medications are included in this feeding category [43]. Complementary and/or replacement feeding is defined as an intake pattern that includes breast milk and solid or semi-solid foods [43]. We included the term “replacement feeding” in this category since formula, foods, or liquids introduced during the first 6 months “replace” breast milk rather than “complement” it [45-47].

### Statistical analysis

All statistical analyses were performed using Statistical Package for Social Sciences version 18.0 (SPSS Inc.; Chicago, IL, USA, 2009). A p-value was set at alpha <0.05 for a two-tailed test. Categorical variables between feeding categories were compared using the Pearson’s chi-square, Yates’ correction for continuity, or Fisher’s exact tests, as appropriate. When normality was confirmed, an independent sample t-test was used to compare continuous variables between the exclusive and the non-exclusive breastfeeding groups. A Mann–Whitney U test was used when normality did not exist. Several variables such as parental ethnicity, place of residence, alcohol consumption, smoking, recreational street drug use, and assisted fertility birth were not evaluated due to heterogeneity and small sample size.

Cronbach’s alpha [48] was used to determine the reliability (internal consistency) of the IIFAS. The regression coefficients were used to estimate the probability of exclusive breastfeeding among the IIFAS score quartile categories. Direct logistic regression analysis identified potential determinants of 6-month exclusive breastfeeding. Multi-collinearity between the variables was examined and, although all variables had normal tolerance (> 0.10), variance inflation factors (VIF) for pre-pregnancy BMI and BMI at 3 months postpartum were high (7.849 and 7.853, respectively), and therefore BMI at 3 months postpartum was excluded from future analyses. The direct binary logistic regression models used only significant variables.

The Hosmer-Lemeshow test [49] assessed the practical utility of each logistic solution, and the results were compared to Nagelkerke R^2^ effect sizes. Final logistic regression analyses on cases (no missing data for IIFAS score, parity, education, and pre-pregnancy BMI) reduced the sample size to 253. Statistical tests indicated that none of the four variables significantly distinguished between cases excluded and included in the regression analysis (Additional file 1).

## Results

The characteristics of women in the present study who breastfed exclusively for 3 and 6 months are presented in Table 1. In general, the women were 31 years of age, were married or living common-law (≥97%), had a university education (≥75%), held paid employment (≥80%), had an annual household income of $70,000 or greater (≥82%), and had been born in Canada (≥82%) (Table 1). Similarly, the majority of fathers had a university degree (≥60%) and had been born in Canada (≥83%).

**Table 1:** **Baseline characteristics of a subsample of participants from the first cohort of Alberta Pregnancy Outcomes and Nutrition (APrON) study in relation to breastfeeding exclusivity for 3 and 6 months**^**†,1,2**^

**Characteristics**	**Exclusive breastfeeding**							
	**For three months postpartum**				**For six months postpartum**			
	**Total**	**No**	**Yes**	**p-value**	**Total**	**No**	**Yes**	**p-value**
		**n = 185**	**n = 217**			**n = 254**	**n = 46**	
**Parental socio-demographic factors**								
Maternal age^3^, *years*	31.0 (6.0)	31.0 (6.0)	31.0 (5.0)	0.539^4^	31.0 (6.0)	31.0 (6.0)	32.0 (5.0)	0.022^4^
Maternal marital Status								
Single/Divorced/Separated	11 (2.7)	6 (3.3)	5 (2.3)	0.761^5^	6 (2.0)	5 (2.0)	1 (2.2)	0.999^5^
Married/Common-law partner	390 (97.3)	178 (96.7)	212 (97.7)		293 (98.0)	248 (98.0)	45 (97.8)	
Maternal education level								
Less than secondary education	114 (28.4)	61 (33.0)	53 (24.4)	0.164^6^	83 (27.7)	75 (29.5)	8 (17.4)	0.007^5^
Completed university undergraduate degree	196 (48.8)	85 (45.9)	111 (51.2)		146 (48.7)	127 (50.0)	19 (41.3)	
Completed university post-graduate degree	92 (22.9)	39 (21.1)	53 (24.4)		71 (23.7)	52 (20.5)	19 (41.3)	
Paid job during pregnancy								
Yes	323 (81.0)	150 (82.0)	173 (80.1)	0.728^7^	242 (81.5)	209 (83.3)	33 (71.7)	0.100^7^
No	76 (19.0)	33 (18.0)	43 (19.9)		55 (18.5)	42 (16.7)	13 (28.3)	
Occupational status during pregnancy^8^								
Full-time	247 (76.7)	117 (78.0)	130 (75.6)	0.704^7^	189 (78.4)	163 (78.4)	26 (78.8)	0.999^5^
Part-time	75 (23.3)	33 (22.0)	42 (24.4)		52 (21.6)	45 (21.6)	7 (21.2)	
Canadian-born mother								
No	62 (15.7)	33 (18.1)	29 (13.6)	0.266^7^	44 (14.9)	37 (14.7)	7 (15.9)	0.820^5^
Yes	334 (84.3)	149 (81.9)	185 (86.4)		251 (85.1)	214 (85.3)	37 (84.1)	
Annual household income, *CAD*								
<20,000	7 (1.8)	5 (2.7)	2 (0.9)	0.277^5^	2 (0.7)	2 (0.8)	0 (0.0)	0.992^5^
20,000-39,000	11 (2.8)	7 (3.8)	4 (1.9)		5 (1.7)	5 (2.0)	0 (0.0)	
40,000-69,000	52 (13.2)	19 (10.4)	33 (15.6)		41 (13.9)	35 (14.1)	6 (13.0)	
70,000-99,000	105 (26.6)	50 (27.3)	55 (25.9)		78 (26.4)	66 (26.5)	12 (26.1)	
≥100,000	220 (55.7)	102 (55.7)	118 (55.7)		169 (57.3)	141 (56.6)	28 (60.9)	
Household size^3^, *n*	2.0 (1.0)	2.0 (1.0)	3.0 (1.0)	0.212^4^	2.0 (1.0)	2.0 (1.0)	3.0 (1.0)	0.135^4^
Paternal education level								
Less than secondary education	113 (40.8)	54 (46.2)	59 (36.9)	0.270^6^	90 (41.1)	79 (43.2)	11 (30.6)	0.055^6^
Completed university undergraduate degree	111 (40.1)	44 (37.6)	67 (41.9)		87 (39.7)	74 (40.4)	13 (36.1)	
Completed university post-graduate degree	53 (19.1)	19 (16.2)	34 (221.3)		42 (19.2)	30 (16.4)	12 (33.3)	
Paternal birth place								
Canada	230 (83.0)	93 (79.5)	137 (85.6)	0.237^7^	181 (82.6)	150 (82.0)	31 (86.1)	0.638^5^
Foreign countries	47 (17.0)	24 (20.5)	23 (14.4)		38 (17.4)	33 (18.0)	5 (13.9)	
**Maternal health information**								
Gravida^3^, *n*	2.0 (1.0)	2.0 (2.0)	2.0 (1.0)	0.124^4^	2.0 (1.0)	2.0 (1.0)	2.0 (2.0)	0.548^4^
Parity								
Primiparous	222 (55.6)	117 (63.6)	105 (48.8)	0.004^7^	175 (58.9)	156 (62.2)	19 (41.3)	0.013^6^
Multiparous	177 (44.4)	67 (36.4)	110 (51.2)		122 (41.1)	95 (37.8)	27 (58.7)	
Planned pregnancy								
Yes	330 (82.3)	144 (78.3)	186 (85.7)	0.069^7^	249 (83.6)	207 (82.1)	42 (91.3)	0.136^5^
No	71 (17.7)	40 (21.7)	31 (14.3)		49 (16.4)	45 (17.9)	4 (8.7)	
Pre-pregnancy weight^3^, *kg*	63.63 (15.89)	65.77 (17.35)	62.66 (14.69)	0.132^4^	63.63 (15.66)	64.09 (15.71)	61.36 (13.40)	0.089^4^
Pre-pregnancy BMI^3,9^, *kg/m*^*2*^	22.87 (5.4)	23.32 (5.7)	22.35 (4.7)	0.016^4^	22.98 (5.4)	22.99 (5.4)	22.12 (5.3)	0.048^4^
Pre-pregnancy BMI categories^9^, *kg/m*^*2*^								
Underweight (≤18.5)	12 (3.1)	3 (1.7)	9 (4.2)	0.266^5^	10 (3.4)	1 (0.7)	9 (5.9)	0.019^5^
Normal (18.6-24.9)	255 (64.9)	113 (62.4)	142 (67.0)		189 (64.5)	86 (61.0)	103 (67.8)	
Overweight (25.0-29.9)	75 (19.1)	38 (21.0)	37 (17.5)		56 (19.1)	33 (23.4)	23 (15.1)	
Obese (≥30)	51 (13.0)	27 (14.9)	24 (11.3)		38 (13.0)	21(14.9)	17 (11.2)	
IIFAS score^10,11^	67.06 (741)	64.42 (7.57)	69.08 (6.62)	<0.001^12^	67.27 (7.59)	66.60 (7.63)	70.67 (6.44)	0.001^12^

CAD: Canadian dollars; BMI: body mass index; IIFAS: Iowa Infant Feeding Attitude Scale.

^†^Even though we evaluated several paternal factors in relation to breastfeeding exclusivity (e.g., paternal marital status, occupation, smoking habits, etc.) since the missing rate was high for many of paternal questions and participation rate was lower among fathers we chose to only include paternal birth place and education in this table.

^1^Values are n (%), unless otherwise noted.

^2^Denominators vary due to missing data.

^3^Median (interquartile range (IQR)).

^4^Mann-Whitney U Test.

^5^Fisher’s exact test.

^6^Pearson’s chi-square.

^7^Yates’ correction for continuity.

^8^Calculated only among mothers who were employed.

^9^BMI was calculated by dividing the weight in kilograms by square of height in meters.

^10^Score ranges from 17-85, with higher scores indicating more positive attitudes toward breastfeeding.

^11^Mean (standard deviation (SD)).

^12^Independent sample t-test.

Women who breastfed exclusively for 6 months were more likely than women who did not breastfeed exclusively for 6 months to hold a university post-graduate degree (41.3% vs. 20.5%), to be multiparous (58.7% vs. 37.8%), and to have a lower pre-pregnancy BMI (22.1 vs. 23.0 kg/m^2^).

### Infant feeding practices

**Figure 2:**
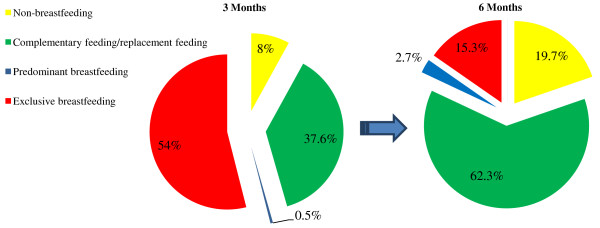
**Infant feeding patterns and transitions between 3 months (n = 402) and 6 months (n = 300) postpartum in a subsample of participants from the first phase of Alberta Pregnancy Outcomes and Nutrition (APrON) study **^**1**,**2**^**.** (legend) ^1^Categories were defined based on the infant feeding guidelines of the World Health Organization^2^. Non-breastfeeding: infants have received no breast milk (directly, expressed, or from a wet nurse) and could be fed any solid/semi-solid foods or liquids including non-human milk. Complementary feeding/replacement feeding: infants have received breast milk (directly, expressed, or from wet nurse) and solid/semi-solid foods, food-based liquids, or non-human milk. Predominant breastfeeding: infants have received breast milk (directly, expressed, or from a wet nurse) as the main source of nourishment, and feeding of certain liquids (water, water-based drinks, and fruit juice), ritual fluids, ORS, drops, and syrups (vitamins, minerals, and medicines) were allowed. Infants in this category have not received anything else especially non-human milk and food-based liquids. Exclusive breastfeeding: infants have received breast milk (directly, expressed, or from a wet nurse) and only ritual fluids, ORS, drops, and syrups (vitamins, minerals, and medicines) were allowed. Infants in this group were not allowed to receive anything else.

**Figure 3:**
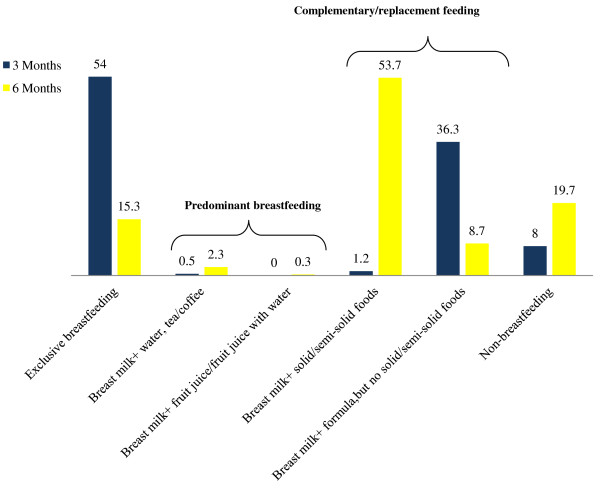
**Detailed breakdown of infant feeding categories during 3 and 6 months postpartum in a subsample of participants from the first phase of Alberta Pregnancy Outcomes and Nutrition (APrON) study**^**1**,**2**^**.** (legend) ^1^Categories were defined based on the infant feeding guidelines of the World Health Organization. ^2^*Non*-*breastfeeding*: requires that infants receive no breast milk (directly, expressed, or from a wet nurse) and could be fed any solid/semi-solid foods or liquids including non-human milk. *Complementary feeding*/*replacement feeding*: requires that infants receive breast milk (directly, expressed, or from a wet nurse) and solid/semi-solid foods, food-based liquids, or non-human milk. *Predominant breastfeeding*: requires that infants receive breast milk (directly, expressed, or from a wet nurse) as the main source of nourishment and allows feeding of certain liquids (water, water-based drinks, and fruit juice), ritual drinks, ORS, drops, and syrups (vitamins, minerals, medicines). Infants in this category are not allowed to receive anything else especially non-human milk and food-based fluids. *Exclusive breastfeeding*: requires that infants receive breast milk (directly, expressed, or from a wet nurse) and only allows intake of drops and syrups (vitamins, minerals, medicines). Infants in this group are not allowed to receive anything else.

In this study, 98.6% of mothers breastfed at some point during the first 6 months postpartum, and 18.3% weaned their infants by 6 months. The prevalence of “exclusive breastfeeding” at 3 and 6 months was 54.0% and 15.3%, respectively (Figure 2). Transitions to the other feeding categories showed an inverse relationship. Instances of “complementary/replacement feeding” rose from 37.6% to 62.3%, non-breastfeeding from 8.0% to 19.7%, and predominant breastfeeding from 0.5% to 2.7% (Figure 3).

By 6 months of age over half (54.7%) of infants received formula and 76.0% were fed other liquids (excluding formula), semi-solids, or solid foods. Between 3 and 6 months 71.0% of breastfed infants had transitioned to solid/semi-solid foods (excluding formula), but there was only a 9.4% increase in “ever formula feeding” (data not shown). Reasons commonly given for discontinuing breastfeeding included: perceived milk insufficiency/breastfeeding problems (50.9%), infants’ unwillingness to suck at the breast (16.4%), self-weaning among infants (10.9%), painful/sore nipples or breasts (9.0%), and fatigue (5.5%).

### Maternal infant feeding knowledge and attitudes

**Figure 4:**
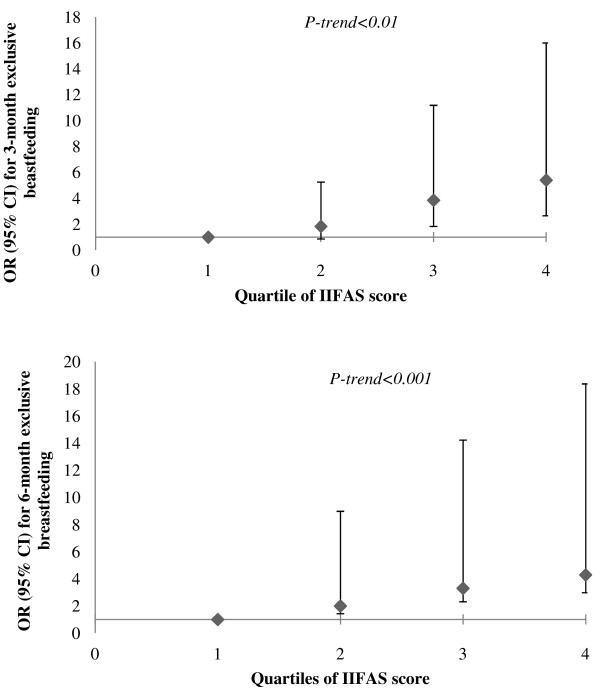
**Adjusted odds ratios (OR) with 95% confidence intervals (CI) for probability of exclusive breastfeeding for 3 and 6 months across the quartiles of Iowa Infant Feeding Attitude Scale (IIFAS) score among a subsample of participants from the first phase of Alberta Pregnancy Outcomes and Nutrition (APrON) study**^**1**,**2**^**.** (legend) ^1^Logisitic regression model of best fit was adjusted for parity (categorical) and pre-pregnancy body mass index (continuous) for 3-month analyses, and for parity (categorical), education (categorical), and pre-pregnancy body mass index (continuous) for 6-month analyses. ^2^IIFAS score range among participants in the 3-month analyses: Quartile 1: 39.00-62.00; Quartile 2: 62.01-67.00; Quartile 3: 67.01-72.00; Quartile 4: 72.01-83.00. IIFAS score range among participants in the 6-month analyses: Quartile 1: 39.00-62.25; Quartile 2: 62.26-67.00; Quartile 3: 67.01-73.00; Quartile 4: 73.01-83.00.

Higher IIFAS scores during pregnancy illustrated the predictive validity of the scores. Higher scores were significantly associated with higher odds of exclusive breastfeeding for 3 months (OR: 1.10, 95% CI: 1.06-1.13; p < 0.001) and 6 months postpartum (OR: 1.08, 95% CI: 1.03-1.14; p = 0.002). The prevalence of any breastfeeding during the first 3 months (OR: 1.43, 95% CI: 1.12-182; p = 0.004) and 6 months (OR: 1.26, 95% CI: 1.08-1.47; p = 004) was significantly higher among mothers who had higher IIFAS scores (p = 0.004) (data not shown). After adjusting binary logistic regression models for potential confounders (i.e., parity, education, pre-pregnancy BMI), women in the highest quartile of the IIFAS score (pro-breastfeeding) were 4.29 times more likely to breastfeed exclusively to 6 months (95% CI: 1.31-14.08) than women in the lowest quartile (pro-formula feeding) (p-trend < 0.001) (Figure 4).

### Comparison of infant feeding knowledge and attitudes

Overall, the IIFAS scores for women in this study were in the neutral range (i.e., 49–69) (means (SD): 67.27(7.59). However, the scores for women who breastfed exclusively for 6 months were significantly higher (mean (SD): 70.67 (6.44); range = 57-82) than for women who did not (mean (SD): 66.60 (7.63); range = 39-83) (p = 0.001) (Table 2).

**Table 2:** **Comparison of statistically significant items in the Iowa Infant Feeding Attitude Scale (IIFAS) between mothers who exclusively breastfed to 6 months and those who did not: Alberta Pregnancy Outcomes and Nutrition (APrON) study **^**1,2**^

**IIFAS items**	**Exclusive breastfeeding for 6 months**		**Difference mean ± SD**
	**No**	**Yes**	
	**Mean ± SD**	**Mean ± SD**	
	**n = 254**	**n = 46**	
Formula feeding is more convenient than breastfeeding (R)^3^	4.03 ± 0.97	4.36 ± 0.82	−0.32 ± 0.16
Formula feeding is the better choice if the mother plans to go back to work (R)^3^	3.74 ± 0.89	4.07 ± 0.78	−0.33 ± 0.15
Mothers who formula feed miss one of the great joys of motherhood	3.29 ± 1.08	3.76 ± 1.03	−0.48 ± 0.18
Women should not breastfeed in public places such as restaurants (R)^3^	4.30 ± 0.83	4.64 ± 0.58	−0.34 ± 0.11
Formula is as healthy for an infant as breast milk (R)^3^	3.43 ± 1.00	3.81 ± 0.80	−0.38 ± 0.14
Breastfeeding is more convenient than formula	3.97 ± 0.96	4.33 ± 0.79	−0.37 ± 0.16

SD: Standard deviation.

^1^Of total 17 IIFAS items, only statistically significant scale items (n = 6) are shown due to copy-right restrictions (Independent sample t-test, 2-sided p-value < 0.05).

^2^IIFAS is comprised of 17 items and responses are on a 5-point Likert scale ranging from “strongly disagree” to “strongly agree”. Total score ranges from 17-85, with higher scores indicating more positive attitudes toward breastfeeding.

^3^(R) indicates reverse-coded items.

Of the 17 items on the IIFAS, the mean values for 6 were significantly higher among mothers who exclusively breastfed to 6 months than among mothers who breastfed non-exclusively (Table 2). In general, mothers who exclusively breastfed to 6 months were less likely to consider formula feeding more convenient than breastfeeding compared to mothers who non-exclusively breastfed (p = 0.04). In addition, mothers who exclusively breastfed for 6 months more strongly disagreed with the statements “women should not breastfeed in public places” (mean (SD): 4.64 ± 0.58 vs. 4.30 ± 0.83; p = 0.002) or “formula is a better option for mothers who plan to return to work” (p = 0.026) than women who did not breastfeed exclusively. Mothers who exclusively breastfed for 6 months were less likely to believe that “formula is as healthy as breast milk”, and they were of the opinion that “mothers who formula feed miss one of the great joys of motherhood” (mean (SD)): 6 ± 1.03 vs. 3.29 ± 1.08; p = 0.009) [30]. Overall, 83.3% of mothers who breastfed exclusively to 6 months and 77.1% of mothers who breastfed non-exclusively to 6 months strongly agreed that breast milk is cheaper (data not shown).

### Follow-up factors

Table 3 shows the results of bivariate analyses for the follow-up maternal and infant characteristics in relation to 3-month and 6-month exclusive breastfeeding. Overall, 59.6% of mothers who exclusively breastfed to 6 months and 45.3% who non-exclusively breastfed had normal BMI values postpartum (p = 0.049).

**Table 3:** **Follow-up postnatal characteristics of a subsample of participants from the first cohort of Alberta Pregnancy Outcomes and Nutrition (APrON) study in relation to breastfeeding exclusivity for 3 and 6 months**^**1,2**^

**Characteristics**	**Exclusive breastfeeding**							
	**For three months postpartum**				**For six months postpartum**			
	**Total**	**No**	**Yes**	**p-value**	**Total**	**No**	**Yes**	**p-value**
		**n = 185**	**n = 217**			**n = 254**	**n = 46**	
**Maternal characteristics**								
Gestational weight gain^3^*, kg*	15.90 (6.6)	15.91 (6.87)	15.45 (6.02)	0.503^4^	15.45 (6.32)	15.59 (6.61)	14.77 (5.34)	0.719^4^
BMI at 12 weeks postpartum^3,5^, *kg/m*^*2*^	24.59 (5.53)	25.18 (6.07)	24.17 (5.19)	0.002^4^	24.47 (5.94)	24.48 (5.95)	23.82 (5.01)	0.048^4^
BMI categories at 12 weeks postpartum^5^, *kg/m*^*2*^								
Underweight (≤18.5)	3 (0.8)	1 (0.5)	2 (1.0)	0.164^6^	3 (1.0)	1 (0.7)	2 (1.3)	0.049^6^
Normal (18.6-24.9)	200 (51.2)	85 (46.7)	115 (55.0)		153(52.8)	63 (45.3)	90 (59.6)	
Overweight (25-29.9)	122 (31.2)	58 (31.9)	64 (30.6)		88 (30.3)	47 (33.8)	41 (27.2)	
Obese (≥30)	66 (16.9)	38 (20.9)	28 (13.4)		46 (15.9)	28 (20.1)	18 (11.9)	
**Infant characteristics**								
Gender								
Female	180 (44.8)	73 (39.5)	107 (49.3)	0.060^7^	140 (46.8)	118 (46.6)	22 (47.8)	0.999^7^
Male	222 (55.2)	112 (60.5)	110 (50.7)		159 (53.2)	135 (53.4)	24 (52.2)	
Gestational age^3,8^, *wks*	39 (2.0)	39.0 (2.0)	39.0 (2.0)	0.591^4^	39.0 (2.0)	39.0 (2.0)	39.0 (2.0)	0.945^4^
Birth weight^9^*, kg*	3.44 (0.6)	3.38 (0.63)	3.49 (0.56)	0.206^3,4^	3.48 (0.46)	3.47 (0.46)	3.53 (0.49)	0.464^10,11^
Weight gain during the first 12 weeks^9,10^, *kg*	2.65 (0.70)	2.66 (0.75)	2.65 (0.66)	0.885^11^	2.63 (0.68)	2.61(0.68)	2.73(0.66)	0.319^11^
Vitamin/mineral supplement intake^12^								
Yes	333 (92.2)	134 (89.3)	199 (94.3)	0.123^7^	184 (76.7)	148 (76.3)	36 (78.3)	0.928^7^
No	28 (7.8)	16 (10.7)	12 (5.7)		56 (23.3)	46 (23.7)	10 (21.7)	

BMI: body mass index.

^1^Values are n (%), unless otherwise noted.

^2^Denominators vary due to missing data.

^3^Median (interquartile range (IQR)).

^4^Mann-Whitney U Test.

^5^BMI was calculated by dividing the weight in kilograms by square of height in meters.

^6^Fisher’s exact test.

^7^Yates’ correction for continuity.

^8^Calculated only among term infants due to initial exclusion of pre-term infants from the analyses.

^9^Calculated only among infants weighing >2.5 kilograms due to initial exclusion of low birth weight infants from the analyses.

^10^Mean (standard deviation (SD)).

^11^Independent-sample t-test.

^12^Calculated only among breast-fed infants.

Among all infants in this study, 53.2% were male, and the infant mean weight gain during the first 3 months was 2.63 (0.68) kg (SD). Among breastfed infants, 23.3% were given no supplements (including vitamin D). In addition, 58.0% of mothers “always” fed their infants on demand and 33.0% avoided “scheduled feeding” (data not shown).

### Predictors of exclusive breastfeeding to 6 months

Table 4 shows the results of direct logistic regression models for potential predictors of breastfeeding exclusivity for 6 months. According to the Wald criterion, three variables made statistically significant contributions to the prediction (IIFAS score, multi-parity, and post-graduate education) and were retained in the final logistic regression model (X^2^ (5, n = 253): 24.50, p <0.001). The logistic regression results did not have convergence problems, and standard errors, as well as bivariate correlation co-efficients, were small.

**Table 4:** Direct (forced-entry) logistic regression analysis of best-fitting model for predictors of 6-month exclusive breastfeeding among a subsample of participants from the first cohort of Alberta Pregnancy Outcomes and Nutrition (APrON) study^*,†^

**Model, predictor**	***β***^***1***^	***SE***^***2***^	**Wald**	**p-value**	***Exp (B)***^***3***^	***95% CI***^***4***^
***Model 1***						
IIFAS score	0.08	0.03	9.76	0.002	1.08	1.03-1.14
Constant	-6.96	1.75	15.86	0.000	0.001	
***Model 2***						
IIFAS score	0.07	0.03	8.78	0.003	1.08	1.03-1.13
Multiparity	0.63	0.35	3.30	0.070	1.88	0.95-3.73
Constant	-7.01	1.75	15.96	0.000	0.001	
***Model 3***						
IIFAS score	0.07	0.03	7.82	0.005	1.08	1.02-1.13
Multiparity	0.77	0.36	4.54	0.033	2.16	1.06-4.39
Completed university undergraduate degree	0.58	0.51	1.29	0.257	1.79	0.66-4.87
Completed university post-graduate degree	1.50	0.53	7.91	0.005	4.46	1.57-12.63
Constant	-7.77	1.92	16.46	0.000	0.000	
***Model4***^5^						
IIFAS score	0.07	0.03	7.70	0.006	1.08	1.02-1.13
Multiparity	0.79	0.37	4.65	0.031	2.21	1.08-4.52
Completed university undergraduate degree	0.52	0.52	1.00	0.318	1.68	0.61-4.63
Completed university post-graduate degree	1.33	0.54	5.93	0.015	3.76	1.30-10.92
Pre-pregnancy BMI^6^	-0.05	0.05	1.30	0.255	0.95	0.87-1.04
Constant	-6.52	2.30	8.05	0.005	0.001	

IIFAS: Iowa Infant Feeding Attitude Scale; BMI: body mass index.

^*^Reference categories include: parity (primiparous); education (less than secondary education).

^†^IIFAS score and pre-pregnancy BMI were entered into models as continuous variables, while multiparity and maternal education were entered as categorical factors.

^1^Coefficient of regression.

^2^Standard error.

^3^Exponential value of β.

^4^95% confidence interval of the exponential value.

^5^Nagelkerke R2 =0.16; Hosmer-Lemeshow test: (X^2^ = 13.31, p = 0.10); positive predictive value = 60.00%; negative predictive value = 84.67%.

^6^BMI was calculated by dividing the weight in kilograms by square of height in meters.

The final regression model suggested that the odds of 6-month exclusive breastfeeding increased 1.08 times for a unit increase in attitude score (95% CI: 1.02-1.13; p = 0.006). Multiparous mothers were 2.21 times more likely to breastfeed exclusively for 6 months (95% CI: 1.08-4.52; p = 0.031) than primiparous mothers. Mothers who held post-graduate degrees were 3.76 times (95% CI = 1.30-10.92) more likely to exclusively breastfeed to 6 months than women who did not have university education (p = 0.015). Although the probability of exclusive breastfeeding to 6 months seemed to be lower among those with higher pre-pregnancy BMIs, this relationship did not achieve statistical significance (p = 0.255).

## Discussion

This is the first longitudinal cohort study to report infant feeding transitions and predictors based on a range of potential maternal factors assessed during both prenatal and postnatal periods. A woman’s choice to breastfeed exclusively was influenced strongly by maternal education, parity, and attitudes toward breastfeeding. Pregnant women who were in the highest quartile of the IIFAS score were more than 4 times more likely to breastfeed exclusively for 3 and 6 months than those who were in the lowest category. We documented a significant shift in feeding patterns of Albertan infants during the first 6 months of life. The diet shifted from essentially nothing but breast milk, formula, or both for the first 3 months of life to a diet of solid foods as the infants reached 6 months of age. Although almost all Albertan infants were breastfed at some point, about half of them were exclusively breastfed for 3 months and 15.3% for 6 months.

The present study shows that breastfeeding exclusivity was undermined by the introduction of solid/semi-solid foods and infant formula. From 3 to 6 months postpartum, a large proportion of infants were fed formula on a regular basis and a higher proportion were fed semi-solid/solid foods. The WHO recommends that children be breastfed for the six months of life without the introduction of complementary foods. In this study, infants passed from exclusive breastfeeding to complementary feeding/replacement feeding without passing through predominant breastfeeding. However, virtually no data are available to form evidence-based recommendations for the introduction of solids in infants who are receiving exclusively or predominantly infant formula.

A feeding shift that excludes a period of predominant breastfeeding, as reported in this study, is consistent with another Canadian study that suggests that Canadian mothers favour complementary feeding/replacement feeding rather than predominant breastfeeding when the infants are at an early age [50]. Although in this study, water and water-based drinks were introduced frequently to infants, the rare practice of predominant breastfeeding requires that no other foods or food-based liquids be introduced.

It is evident that there is room for improvement in Canadian infant feeding practices. More European infants transition from exclusive breastfeeding to predominant breastfeeding and fewer receive complementary foods including formula during the first weeks of life [44,51-55]. The differences in feeding patterns between European and North American infants may be explained by the more aggressive marketing of infant formula in North America than in Europe, or they may be a reflection of cultural differences between the two regions [50]. Compared to women in other developed countries, women in North America tend to breastfeed for shorter periods [28,56,57]. Why Canadian women engage in such early complementary feeding behaviours is not clearly understood; it may express their intentions to replace breast milk or prematurely wean their infants [45,58,59] or it may represent unresolved feeding problems, all of which contribute to shorter breastfeeding periods [60]. The breastfeeding rates of Albertan mothers in this study are similar to those reported by national Canadian surveys [25,26] and other developed nations [61]. In industrialized countries in general, the duration of exclusive breastfeeding is short, with the notable exception of the Nordic European region [61-64].

While more than 20% of breastfeeding mothers in this study did not provide vitamin D supplements to their infants, about half of exclusively breastfeeding mothers in another national survey gave their infants vitamin D supplements [24]. Exclusively breastfed infants living in Canada who do not take vitamin D supplements are at greater risk of vitamin D deficiency and insufficiency as well as rickets [21,65,66]. In Canada, a daily supplement of vitamin D (400 IU) is recommended for all breastfed full-term infants as a way to compensate for minimal exposure to sunlight [21,64,65], to promote health and to prevent deficiency.

More than half of the mothers in this study discontinued breastfeeding because of their perceptions of milk inadequacy or other breastfeeding problems. A perception of milk inadequacy is a commonly cited reason for breastfeeding cessation [67] and may have a strong psychological component that may be rooted in low self-esteem during the early postpartum period [68]. While 1-5% of women in a population may have an insufficient milk supply, as many as 50% of women believe they do [68]. Mothers who overcame breastfeeding problems developed a greater sense of self-efficacy that was associated with continued breastfeeding [69], and future breastfeeding programs should focus on a mother’s disbelief in her ability and sense of self-efficacy to breastfeed.

This is the first Canadian study to use the psychometric results of the IIFAS to evaluate women’s infant feeding knowledge and attitudes in relation to 6-month exclusive breastfeeding behaviours. In the present study, mothers who had higher IIFAS scores had an increasingly higher probability of 3-month and 6-month exclusive breastfeeding. These results suggest that pregnant women’s attitudes toward breastfeeding may be a good indicator of intentions and may shape future infant feeding behaviours. In addition, a large proportion of women in this study had neutral attitudes toward breastfeeding, as did about half the mothers who fed their infants formula during the first 6 months postpartum. A neutral attitude toward breastfeeding during the prenatal period suggests that women’s feeding intentions may not be fully formed. This neutrality toward feeding practices may be used by present health professionals as an opportunity to deliver maternal educational programs.

Similar to a recent review [70], this study demonstrated that IIFAS can be a valid and reliable tool to measure infant feeding attitudes among prenatal women. This self-report tool has several advantages over similar instruments, including simplicity, ease of use, simple wording, and applicability to a wide range of groups [70-73]. However, more studies are needed to evaluate the predictive validity of the IIFAS among diverse population groups. Since the IIFAS score was only 3 units higher among mothers who exclusively breastfed compared to those who did not, and the scores were slightly different between mothers practicing exclusive breastfeeding and those who breastfed non-exclusively, future evaluations of this tool in different settings with diverse populations are warranted.

Several international [74,75] and Canadian studies [23,28,76-80] also found that maternal socio-demographic and lifestyle factors determined maternal infant feeding behaviours. Similar to other Canadian studies, the present study found that maternal education was the strongest predictor of breastfeeding exclusivity and that mothers who hold post-graduate degrees are 3.5 times more likely to breastfeed exclusively to 6 months, compared to those without a post-secondary education [23,24,28,80,81]. Maternal education was associated with breastfeeding initiation and continuation as well as with types of liquids and solids fed to infants in studies from Canada [24,28,80,81] and from other countries [31,74,75,82]. Mothers with higher education were more likely to have well-informed infant feeding decisions and were more receptive to positive health messages including the benefits of breastfeeding [23,71].

In the present study, mothers with previous children were more than twice as likely as first-time mothers to breastfeed exclusively for 6 months. Similar to our findings, previous studies suggested a dose–response relationship between parity, and breastfeeding initiation and exclusivity among mothers of both singleton and twin infants [23,26,36,74,83]. Another study found that first-born children were more likely to be weaned early and introduced to cow’s milk and formula early [84]. Multiparous women have higher self-confidence, self-efficacy, and infant feeding knowledge gained through earlier breastfeeding experiences and were more likely to breastfeed exclusively for 6 months [36].

This study has several strengths. This is the first Canadian study to use the psychometric properties present in results from the IIFAS. This is also the first prospective cohort study in Alberta to report on infant feeding transitions and pre- and postnatal predictors of breastfeeding. Another strength of this study is its use of the latest WHO infant feeding definitions [43] to evaluate feeding practices from birth using a prospective cohort design. As well, in this study a short-term recall (3-month interval) was used rather than a long-term recall (up to 5-year interval). The short recall period likely decreased the recall bias and increased the overall reliability of results.

However, limitations of this study should also be noted. First, this study has a recruitment bias; there is an over-representation of mothers from a higher socio-economic status, and this bias may limit the generalizability of the results. Similar problems were reported in other infant feeding studies conducted in industrialized countries due to the possibility of self-selection bias among health-attentive participants who volunteer for these studies [85,86]. Second, the homogeneity of the sample limited our ability to compare attitudes of different cultural and socio-economic groups toward breastfeeding since the groups was comprised of Caucasian women with a high income status. The lack of diversity in our sample resulted in no significant associations between a wide range of parental/infant variables and infant feeding practices. Third, there is a risk that social pressure to breastfeed influenced mothers who are susceptible to that pressure to over-report breastfeeding rates. Finally, this study would have benefited from information on partners’ support and attitudes toward breastfeeding.

## Conclusions

Despite the high proportion of breastfed infants, only 15.3% of infants were breastfed exclusively for 6 months. Breastfeeding promotion programs in Alberta seem to be successful in achieving high rates of breastfeeding initiation; however, a shift in focus is required to promote breastfeeding exclusivity. Given striking disparities in infant feeding practices across provinces in Canada [23,24], closer scrutiny of infant feeding practices may be required to better understand the determinants of feeding behaviors.

Given that maternal knowledge about and attitudes toward infant feeding are malleable and may be changed through education and behavioural interventions, a good understanding of behavioural determinants is needed to design targeted interventions that address women’s misconceptions about formula feeding and milk insufficiency, with a special emphasis on young, first-time mothers. Also, policy makers should be informed of the need to make provision of more nursing rooms a priority to encourage breastfeeding.

## Competing interests

The authors declare that they have no competing interests arising from this research.

## Authors’ contributions

This study was part of a MSc thesis submitted by MJ to the University of Alberta, Canada. MJ and APF conceptualized the study. MJ analyzed the data. APF guided and supervised the study, assisted in the interpretation of findings, and drafted the manuscript. KM and NDW helped with interpretation of data and final analyses. RCB provided comments on all drafts of the manuscript. All authors read and approved the final manuscript.

## Pre-publication history

The pre-publication history for this paper can be accessed here:

http://www.biomedcentral.com/1471-2431/13/77/prepub

## Supplementary Material

Additional file 1 Comparison of cases excluded from the regression models due to missing values for any of the potential predictors of exclusive breastfeeding and those included in the final regression models: Alberta Pregnancy Outcomes and Nutrition (APrON) study. Click here for file

## References

[B1] Kramer MS Chalmers B Hodnett ED Sevkovskaya Z Dzikovich I Shapiro S Collet JP Vanilovich I Mezen I Ducruet T Shishko G Zubovich V Mknuik D Gluchanina E Dombrovskiy V Ustinovitch A Kot T Bogdanovich N Ovchinikova L Helsing E PROBIT Study Group (Promotion of Breastfeeding Intervention Trial):Promotion of Breastfeeding Intervention Trial (PROBIT): a randomized trial in the Republic of Belarus JAMA 2001 285 4 413 420 10.1001/jama.285.4.413 11242425

[B2] Kramer MS Aboud F Mironova E Vanilovich I Platt RW Matush L Igumnov S Fombonne E Bogdanovich N Ducruet T Collet JP Chalmers B Hodnett E Davidovsky S Skugarevsky O Trofimovich O Kozlova L Shapiro S Promotion of Breastfeeding Intervention Trial (PROBIT) Study Group:Breastfeeding and child cognitive development: new evidence from a large randomized trial Arch Gen Psychiatry 2008 65 5 578 584 10.1001/archpsyc.65.5.578 18458209

[B3] Horta BL Bahl R Martines JC Victora CG Evidence on the long-term effects of breastfeeding.Systematic reviews and meta-analysis 2007 Geneva: World Health Organization

[B4] Ip S Chung M Raman G Chew P Magila N DeVine D Trikalinos T Lau J Breastfeeding and maternal and child health outcomes in developed countries 2007 Rockville: MD: Agency for Healthcare Research and Quality AHRQ Publication No.: 07-E007 PMC4781366 17764214

[B5] Gartner LM Morton J Lawrence RA Naylor AJ O’Hare D Schanler RJ Eidelman AI American Academy of Pediatrics Section on Breastfeeding: Breastfeeding and the use of human milk Pediatrics 2005 115 496 506 15687461 10.1542/peds.2004-2491

[B6] U.S. Department of Health and Human Services The Surgeon General’s call to action to support breastfeeding 2011 Washington, DC: U.S: Department of Health and Human Services, Office of the Surgeon General 10.3945/an.111.000968 PMC3226390 22332095

[B7] Fewtrell MS The long-term benefits of having been breast-fed CurrPaediatr 2004 14 97 103

[B8] Duijts L Jaddoe VW Hofman A Moll HA Prolonged and exclusive breastfeeding reduces the risk of infectious diseases in infancy Pediatrics 2010 126 1 e18 e25 Epub 2010 Jun 21 10.1542/peds.2008-3256 20566605

[B9] Binns CW Lee M Scott JA The fetal origins of disease hypothesis: public health implications for the Asia-Pacific region Asia Pac J Public Health 2001 13 2 68 73 10.1177/101053950101300202 12597501

[B10] Heinig MJ Dewey KG Health effects of breast feeding for mothers: a critical review Nutr Res Rev 1997 10 1 35 56 10.1079/NRR19970004 19094257

[B11] Arenz S Ruckerl R Koletzko B von Kries R Breast-feeding and childhood obesity: a systematic review Int J Obes Relat Metab Disord 2004 28 1247 1256 10.1038/sj.ijo.0802758 15314625

[B12] Owen CG Martin RM Whincup PH Smith GD Cook DG Does breastfeeding influence risk of type 2 diabetes in later life? A quantitative analysis of published evidence Am J Clin Nutr 2006 84 1043 1054 17093156 10.1093/ajcn/84.5.1043

[B13] Bhandari N Bahl R Mazumdar S Martines J Black RE Bhan MK Infant Feeding Study Group:Effect of community-based promotion of exclusive breastfeeding on diarrhoeal illness and growth: a cluster randomised controlled trial Lancet 2003 361 9367 1418 1423 10.1016/S0140-6736(03)13134-0 12727395

[B14] Kramer MS Kakuma R The optimal duration of exclusive breastfeeding: a systematic review Adv Exp Med Bio 2004 554 63 77 Review 15384567 10.1007/978-1-4757-4242-8_7

[B15] Baker JL Gamborg M Heitmann BL Lissner L Sørensen TI Rasmussen KM Breastfeeding reduces postpartum weight retention Am J Clin Nutr 2008 88 6 1543 1551 10.3945/ajcn.2008.26379 19064514

[B16] World Health Organization Infant and young child nutrition 1993 Geneva: WHO

[B17] World Health Organization (WHO) Up to what age can a baby stay well nourished by just being breastfed? 2012 Geneva, Switzerland: WHO Available from: http://www.who.int/features/qa/21/en/

[B18] Chien PF Howie PW Breast milk and the risk of opportunistic infection in infancy in industrialized and non-industrialized settings Adv Nutr Res 2001 10 69 104 11795054 10.1007/978-1-4615-0661-4_4

[B19] Kwan ML Buffler PA Abrams B Kiley VA Breastfeeding and the risk of childhood leukemia: a meta-analysis Public Health Rep 2004 119 521 535 10.1016/j.phr.2004.09.002 15504444 PMC1497668

[B20] Bachrach VR Schwarz E Bachrach LR Breastfeeding and the risk of hospitalization for respiratory disease in infancy: a meta-analysis Arch Pediatr Adolesc Med 2003 157 237 243 10.1001/archpedi.157.3.237 12622672

[B21] Health Canada Expert Advisory Panel on Exclusive Breastfeeding. Exclusive breastfeeding duration- 2004 Health Canada recommendation 2004 Ottawa: Health Canada Available from: http://www.brandonrha.mb.ca/export/sites/brandonrha/galleries/pdf/Having_a_Baby/Canada_Health_Breastfeeding.pdf

[B22] Canadian Paediatric Society Dietitians of Canada and Health Canada: Nutrition for healthy term infants-Statement of the Joint Working Group 2005 Ottawa: Minister of Public Works and Government Services Available from: http://www.hc-sc.gc.ca/fn-an/nutrition/infant-nourisson/recom/index-eng.php

[B23] Al-Sahab B Lanes A Feldman M Tamim H Prevalence and predictors of 6-month exclusive breastfeeding among Canadian women: a national survey BMC Pediatr 2010 10 20 10.1186/1471-2431-10-20 20377899 PMC2858135

[B24] Millar WJ Maclean H Breastfeeding practices Health Rep 2005 16 23 3116190322

[B25] Statistics Canada Health indicator profile, annual estimates, by age group and sex, Canada, provinces, territories, health regions (2011 boundaries) and peer groups, occasional 2011 Canada: CANSIM database Available from: http://www5.statcan.gc.ca/cansim/a01?lang=eng

[B26] Public Health Agency of Canada What mothers say: the Canadian Maternity Experiences Survey 2009 Ottawa, Canada: PHAC Available from: http://www.phac-aspc.gc.ca/rhs-ssg/pdf/survey-eng.pdf

[B27] Friel JK Isaak CA Hanning R Miller A Complementary food consumption of Canadian infants Open Nutr J 2009 3 11 16 10.2174/1874288200903010011

[B28] Dubois L Girard M Social determinants of initiation, duration and exclusivity of breastfeeding at the population level: The results of the longitudinal study of child development in Quebec (ELDEQ 1998–2002) Can J Public Health 2003 94 300 305 12873091 10.1007/BF03403610 PMC6979909

[B29] Dungy C Losch M Russell D Maternal attitudes as predictors of infant feeding decisions J Assoc Acad Minor Phys 1994 5 4 159 164 7812084

[B30] de la Mora A Russell D Dungy C Losch M Dusdieker L The Iowa Infant Feeding Attitude Scale: Analysis of reliability and validity J Appl Soc Psychol 1999 29 11 2362 2380 10.1111/j.1559-1816.1999.tb00115.x

[B31] Scott JA Landers M Hughes R Factors associated with breastfeeding at discharge and duration of breastfeeding amongst two populations of Australian women J Paediatr Child Health 2001 37 254 261 10.1046/j.1440-1754.2001.00646.x 11468040

[B32] Dungy CI McInnes RJ Tappin DM Wallis AB Oprescu F Infant feeding attitudes and knowledge among socioeconomically disadvantaged women in Glasgow Matern Child Health J 2008 12 3 313 322 Epub 2007 Aug 10 10.1007/s10995-007-0253-9 17690964

[B33] Ajzen I Fishbein M Attitude-behaviour relations: a theoretical analysis and review of empirical research Psychol Bull 1977 84 888 918

[B34] Elder J Ayala G Harris S Theories and intervention approaches to health-behaviour change in primary care Am J Prev Med 1999 17 275 284 10.1016/S0749-3797(99)00094-X 10606196

[B35] Losch M Dungy C Russell D Dusdieker L Impact of attitudes on maternal decision regarding infant feeding J Pediatr 1995 126 507 514 10.1016/S0022-3476(95)70342-X 7699527

[B36] Amin T Hablas H Al Qader AA Determinants of initiation and exclusivity of breastfeeding in Al Hassa, Saudi Arabia Breastfeed Med 2011 6 2 59 68 Epub 2010 Oct 29 10.1089/bfm.2010.0018 21034163

[B37] Fomon S Infant feeding in the 20th century: formula and beikost J Nutr 2001 131 2 409S 420S 11160571 10.1093/jn/131.2.409S

[B38] Kaplan BJ Giesbrecht GF Leung B Field CJ Dewey D Bell RC Manca RC Manca D O'Beirne M Johnston DW Pop VJ Singhal N Gagnon L Bernier FB Eliasziw M McCargar L Kooistra L Farmer A Cantell M Goonewardene L Casey LM Letourneau N Martin J The Alberta Pregnancy Outcomes and Nutrition (APrON) cohort study: Rationale and Methods. Maternal and Child Nutrition Matern Child Nutr 2012 [Epub ahead of print] 10.1111/j.1740-8709.2012.00433.x PMC6860282 22805165

[B39] Ho YJ McGrath JM A review of the psychometric properties of breastfeeding assessment tools J Obstet Gynecol Neonatal Nurs 2010 39 4 386 400 10.1111/j.1552-6909.2010.01153.x 20629926

[B40] Fox MK Devaney B Reidy K Razafindrakoto C Ziegler P Relationship between portion size and energy intake among infants and toddlers: evidence of self-regulation J Am Diet Assoc 2006 106 1 Suppl 1 S77 S83 10.1016/j.jada.2005.09.039 16376632

[B41] Fox MK Reidy K Karwe V Ziegler P Average portions of foods commonly eaten by infants and toddlers in the United States J Am Diet Assoc 2006 106 1 Suppl 1 S66 S76 10.1016/j.jada.2005.09.042 16376631

[B42] Fox MK Reidy K Novak T Ziegler P Sources of energy and nutrients in the diets of infants and toddlers J Am Diet Assoc 2006 106 1 Suppl 1 S28 S42 10.1016/j.jada.2005.09.034 16376628

[B43] World Health Organization Indicators for assessing infant and young child feeding practices: conclusion of a consensus meeting held 6–8 November 2007 in Washington DC, USA 2008 Geneva, Switzerland: World Health Organization

[B44] World Health Organization (WHO) Exclusive breastfeeding 2012 Geneva, Switzerland: WHO Available from: http://www.who.int/nutrition/topics/exclusive_breastfeeding/en/index.html

[B45] Aarts C Kylberg E Hörnell A Hofvander Y Gebre-Medhin M Greiner T How exclusive is exclusive breastfeeding? A comparison of data since birth with current status data Int J Epidemiol 2000 29 6 1041 1046 10.1093/ije/29.6.1041 11101545

[B46] Cohen RJ Brown KH Canahuati J Rivera LL Dewey KG Effects of age of introduction of complementary foods on infant breast milk intake, total energy intake, and growth: a randomised intervention study in Honduras Lancet 1994 344 8918 288 293 10.1016/S0140-6736(94)91337-4 7914260

[B47] Dewey KG Cohen RJ Brown KH Rivera LL Age of introduction of complementary foods and growth of term, low-birth-weight, breast-fed infants: a randomized intervention study in Honduras Am J Clin Nutr 1999 69 4 679 686 10197569 10.1093/ajcn/69.4.679

[B48] Cronbach LJ Coefficient alpha and the internal structure of tests Psychometrika 1951 16 3 297 334 10.1007/BF02310555

[B49] Hosmer DW Lemeshow S Applied logistic regression 1989 New. York, NY: Wiley

[B50] Haiek LN Gauthier DL Brosseau D Rocheleau L Understanding breastfeeding behavior: rates and shifts in patterns in Québec J Hum Lact 2007 23 1 24 31 10.1177/0890334406297278 17293548

[B51] Cattaneo A Borgnolo G Simon G Breastfeeding by objectives Eur J Public Health 2001 11 397 401 10.1093/eurpub/11.4.397 11766480

[B52] Heiberg Endresen E Helsing E Changes in breastfeeding practices in Norwegian maternity wards: national surveys 1973, 1982 and 1991 Acta Paediatr 1995 84 719 724 10.1111/j.1651-2227.1995.tb13744.x 7549286

[B53] Kind C Schubiger G Schwarz U Tonz O Provision of supplementary fluids to breast fed infants and later breast feeding success Adv Exp Med Biol 2000 478 347 354 11065084 10.1007/0-306-46830-1_29

[B54] Wyss C Koschorke M Declercq C Monitoring baby-friendliness in certified maternity facilities and clinics and hospitals seeking “Baby-Friendly” certification (Baby-Friendly Hospital Initiative).Report 2001 Abbreviated Version, An analysis on behalf of the Swiss Foundation for the Promotion of Breastfeeding and the Swiss Committee for UNICEF 2003 Basel Institute for Social and Preventive Medicine 13

[B55] Giovannini M Riva E Banderali G Salvioni M Radaelli G Agostoni C Exclusive versus predominant breastfeeding in Italian maternity wards and feeding practices through the first year of life J Hum Lact 2005 21 259 265 10.1177/0890334405277898 16113014

[B56] Centers for Disease Control and Prevention Breastfeeding practices: results from the 2003 National Immunization Survey 2011 Atlanta, GA: CDC Available from: http://eclkc.ohs.acf.hhs.gov/hslc/tta-system/ehsnrc/Early%20Head%20Start/health-safety-nutrition/breastfeeding/BreastfeedingPra.htm

[B57] Health Canada Canadian Perinatal Health Report 2003 Ottawa: Minister of Public Works and Government Services Canada 227 Available from: http://publications.gc.ca/site/eng/252200/publication.html

[B58] Chen CH Chi CS Maternal intention and actual behavior in infant feeding at one month postpartum Acta Paediatr Taiwan 2003 44 3 140 144 14521018

[B59] Hornell A Hofvander Y Kylberg E Solids and formula: association with pattern and duration of breastfeeding Pediatrics 2001 107 E38 10.1542/peds.107.3.e38 11230619

[B60] Gray-Donald K Kramer MS Munday S Leduc DG Effect of formula supplementation in the hospital on the duration of breast-feeding: a controlled clinical trial Pediatrics 1985 75 514 518 3883306

[B61] Yngve A Sjostrom M Breastfeeding in countries of the European Union and EFTA: current and proposed recommendations, rationale, prevalence, duration and trends Public Health Nutr 2001 4 631 645 11683556 10.1079/phn2001147

[B62] Fleischer Michaelsen K Weaver L Branca F Robertson A Feeding and nutrition of infants and young children: guidelines for the WHO European region, with emphasis on the former Soviet countries 2000 Geneva, Switzerland: WHO Europe, UNICEF 288 Available from: http://www.euro.who.int/__data/assets/pdf_file/0004/98302/WS_115_2000FE.pdf

[B63] United Nations The state of the world’s children, statistical tables 2006 New York: USA: UNICEF Available from: http://www.unicef.org/sowc07/docs/sowc07.pdf

[B64] World Health Organization Development of a global strategy on infant and young child feeding 2001 Geneva, Switzerland: WHO 104 Available from: http://www.who.int/nutrition/publications/infantfeeding/9241562218/en/

[B65] Department of National Health and Welfare The Canadian mother and child. The Division 1967 2 Ottwa: Queen’s printer

[B66] Health Canada Expert Advisory Panel on Exclusive Breastfeeding. Vitamin D supplementation for breastfed infants: questions and answers for professional 2012 Ottawa, Canada: Health Canada Available from: http://www.hc-sc.gc.ca/fn-an/surveill/nutrition/commun/prenatal/duration-duree-eng.php

[B67] Hector D King L Webb K Heywood P Factors affecting breastfeeding practices: applying a conceptual framework N S W Public Health Bull 2005 16 3–4 52 55 16106273 10.1071/nb05013

[B68] Dennis C Breastfeeding Initiation and Duration: A 1990–2000 Review J Obstet Gynecol Neonat Nurs 2001 31 1 12 32 10.1111/j.1552-6909.2002.tb00019.x 11843016

[B69] DiGirolamo A Thompson N Martorell R Fein S Grummer-Strawn L Intention or experience? Predictors of continued breastfeeding Health EducBehav 2005 32 2 208 226 10.1177/1090198104271971 15749967

[B70] Chambers JA McInnes RJ Hoddinott P Alder EM A systematic review of measures assessing mothers' knowledge, attitudes, confidence and satisfaction towards breastfeeding Breastfeed Rev 2007 15 3 17 25 18062138

[B71] Li R Hsia J Fridinger F Hussain A Benton-Davis S Grummer-Strawn L Public beliefs about breastfeeding policies in various settings J Am Diet Assoc 2004 104 7 1162 1168 10.1016/j.jada.2004.04.028 15215778

[B72] Spurles PK Babineau J A qualitative study of attitudes toward publicbreastfeeding among young Canadian men and women J Hum Lact 2011 27 2 131 137 Epub 2010 Dec 31 10.1177/0890334410390044 21196495

[B73] Scott JA Mostyn T Greater Glasgow Breastfeeding Initiative Management Team Women’s experiences of breastfeeding in a bottle-feeding culture J Hum Lact 2003 19 3 270 277 10.1177/0890334403255225 12931778

[B74] Lande B Andersen LF Baerug A Trygg KU Lund-Larsen K Veierød MB Bjørneboe GE Infant feeding practices and associated factors in the first six months of life: the Norwegian infant nutrition survey Acta Paediatr 2003 92 2 152 161 12710639 10.1111/j.1651-2227.2003.tb00519.x

[B75] Dulon M Kersting M Schach S Duration of breastfeeding and associated factors in Western and Eastern Germany Acta Paediatr 2001 90 8 931 935 11529545

[B76] Nolan L Goel V Sociodemographic factors related to breastfeeding in Ontario: Results from the Ontario Health Survey Can J Public Health 1995 86 309 312 8556676

[B77] Maclean HM Millar W Breastfeeding in Canada: A review and update 1999 Ottawa: Publications, Health Canada

[B78] Williams PL Innis SM Vogel A Breastfeeding and weaning practices in Vancouver Can J Public Health 1996 87 231 236 8870300

[B79] Yang Q Wen SW Dubois L Chen Y Walker MC Krewski D Determinants of breast-feeding and weaning in Alberta. Canada J Obstet Gynaecol Can 2004 26 11 975 981 15560860 10.1016/s1701-2163(16)30419-4

[B80] Sheehan D Krueger P Watt S Sword W Bridle B The Ontario Mother and Infant Survey: breastfeeding outcomes J Hum Lact 2001 17 3 211 219 10.1177/089033440101700304 11847986

[B81] Health Canada Duration of exclusive breastfeeding in Canada: key statistics and graphics (2007–2008) 2012 Ottawa, Canada: Health Canada Available from: http://www.hc-sc.gc.ca/fn-an/surveill/nutrition/commun/prenatal/duration-duree-eng.php

[B82] Schiess S Grote V Scaglioni S Luque V Martin F Stolarczyk A Vecchi F Koletzko B European Childhood Obesity Project:Introduction of complementary feeding in 5 European countries J Pediatr Gastroenterol Nutr 2010 50 92 98 10.1097/MPG.0b013e31819f1ddc 19543110

[B83] Yokoyama Y Wada S Sugimoto M Katayama M Saito M Sono J Breastfeeding rates among singletons, twins and triplets in Japan: a population-based study Twin Res Hum Genet 2006 9 298 302 10.1375/twin.9.2.298 16611502

[B84] Ummarino M Albano F De Marco G Mangani S Aceto B Ummarino D Correra A Giannetti E De Vizia B Guarino A Short duration of breastfeeding and early introduction of cow’s milk as a result of mothers’ low level of education Acta Paediatr 2003 91 S12 S17 10.1111/j.1651-2227.2003.tb00641.x 14599037

[B85] O'Herlihy BP Breast feeding: incidence and influences Ir Med J 1978 71 12 404 407 700979

[B86] Hurley M Fogarty J A study of infant feeding practices in Ireland 2000 Dublin: Eastern Health Board

