# Supplementary material for: Predictors of exclusive breastfeeding: observations from the Alberta pregnancy outcomes and nutrition (APrON) study
Source: BMC Pediatr. 2013 May 16;13:77. doi: 10.1186/1471-2431-13-77 (PMC3660294; doi:10.1186/1471-2431-13-77)
Supplement: Additional file 1 — Comparison of cases excluded from the regression models due to missing values for any of the potential predictors of exclusive breastfeeding and those included in the final regression models: Alberta Pregnancy Outcomes and Nutrition (APrON) study. [file 1471-2431-13-77-S1.doc]

**Additional File 1.** Comparison of cases excluded from the regression models due to missing values for any of the potential predictors of exclusive breastfeeding and those included in the final regression models: Alberta Pregnancy Outcomes and Nutrition (APrON) study

| **Variables** | | **Excluded from regression analyses**  n=47 | **Included in regression analyses**  n=253 | **p-value** |
| --- | --- | --- | --- | --- |
| IIFAS score^1^ | | 62.67 (5.77) | 67.32 (7.60) | 0.292^2^ |
| Parity^3^ | |  |  |  |
|  | Primiparous | 31 (70.5) | 144 (56.9) | 0.129^4^ |
|  | Multiparous | 13 (29.5) | 109 (43.1) |  |
| Maternal education^3^ | |  |  | 0.567^5^ |
|  | Less than secondary education | 16 (34.0) | 67 (26.5) |
|  | Completed university undergraduate degree | 21 (44.7) | 125 (49.4) |
|  | Completed university post-graduate degree | 10 (21.3) | 61 (24.1) |
| Pre-pregnancy BMI^6,7^, *kg/m^2^* | | 22.40 (5.12) | 22.98 (5.55) | 0.575^8^ |

IIFAS: Iowa Infant Feeding Attitude Scale; BMI: Body mass index

^1^Mean (standard deviation (SD))

^2^Based on independent sample t-test

^3^n (%)

^4^Based on Yates’ correction for continuity

^5^Based on Pearson’s chi-square test

^6^Median (interquartile range (IQR))

^7^Body mass index was calculated by dividing the weight in kilograms by square of height in meters

^8^Based on Mann-Whitney U test
